# A Systematic Review of Attention Biases in Opioid, Cannabis, Stimulant Use Disorders

**DOI:** 10.3390/ijerph15061138

**Published:** 2018-06-01

**Authors:** Melvyn Zhang, Jiangbo Ying, Tracey Wing, Guo Song, Daniel S. S. Fung, Helen Smith

**Affiliations:** 1 National Addiction Management Service, Institute of Mental Health, Singapore 539747, Singapore; song_guo@imh.com.sg; 2 Family Medicine & Primary Care, Lee Kong Chian School of Medicine, Nanyang Technological University Singapore, Singapore 308232, Singapore; h.e.smith@ntu.edu.sg; 3 National Psychiatry Residency Program, National Healthcare Group, Singapore 539747, Singapore; yingjiangbo@gmail.com (J.Y.); tracey.wing@mohh.com.sg (T.W.); 4 Institute of Mental Health, Singapore 539747, Singapore; Daniel_fung@imh.com.sg

**Keywords:** attention bias, cognitive bias, addiction, opioids, cannabis, stimulants

## Abstract

*Background*: Opiates, cannabis, and amphetamines are highly abused, and use of these substances are prevalent disorders. Psychological interventions are crucial given that they help individuals maintain abstinence following a lapse or relapse into substance use. Advances in experimental psychology have suggested that automatic attention biases might be responsible for relapse. Prior reviews have provided evidence for the presence of these biases in addictive disorders and the effectiveness of bias modification. However, the prior studies are limited, as they failed to include trials involving participants with these prevalent addictive disorders or have failed to adopt a systematic approach in evidence synthesis. *Objectives*: The primary aim of this current systematic review is to synthesise the current evidence for attention biases amongst opioid use, cannabis use, and stimulant use disorders. The secondary aim is to determine the efficacy of attention bias modification interventions and other addictions related outcomes. *Methods*: A search was conducted from November 2017 to January 2018 on PubMed, MEDLINE, Embase, PsycINFO, Science Direct, Cochrane Central, and Scopus. The selection process of the articles was in accordance with the Preferred Reporting Items for Systematic Reviews and Meta-Analysis guidelines. A qualitative synthesis was undertaken. Risk of bias was assessed using the Cochrane Risk of Bias tool. *Results*: Six randomised trials were identified. The evidence synthesized from these trials have provided strong evidence that attentional biases are present in opioid and stimulant use disorders. Evidence synthesis for other secondary outcome measures could not be performed given the heterogeneity in the measures reported and the limited number of trials. The risk of bias assessment for the included trials revealed a high risk of selection and attrition bias. *Conclusions*: This review demonstrates the potential need for interventions targeting attention biases in opiate and cocaine use disorders.

## 1. Introduction

The United Nations Office on Drugs and Crime (UNODC) reported that, in 2015, at least 29.5 million individuals were afflicted with a substance use disorder globally [1]. Opiates, cannabis, and amphetamines are highly abused [1]. Substance use disorders are associated with multiple comorbidities. Treatment of substance use disorders involve both pharmacological and psychological approaches [2]. Pharmacological options remain limited, with only opiate substitution therapies available for opioid use disorder in some countries. The main pharmacological options for cannabis and stimulant use disorders are limited to the use of symptomatic medications, such as benzodiazepines for agitation, or antipsychotics for psychotic symptoms [3]. Psychological interventions are crucial given that they help individuals maintain abstinence following a lapse or relapse into substance use. Psychological interventions commonly used include cognitive behavioural therapy for relapse prevention, contingency management, and more recently, mindfulness-based relapse prevention [2]. A prior review which examined 34 randomised controlled trials involving a total of 2340 alcohol and substance use individuals reported that cognitive behavioural therapy was useful, with an effect size of 0.45 (Cohen D) [4]. Cognitive behavioural therapies for substance use disorder typically equip individuals with skills to help them deal with both internal and external triggers, cognitive distortions, and decision delay skills relating to substance use. While therapies such as cognitive behavioural therapy have proven to be effective, other studies [5] have reported that 40–50% of individuals do relapse within a year of successful treatment, and 70% of individuals relapse within three years. The high relapse rate following a moderately effective intervention is of concern and suggests that interventions may not have adequately addressed all the issues leading to either a lapse or relapse. 

Advances in experimental psychology has led to the “dual-process” model of addiction, which proposes there being an imbalance between the impulsive (limbic) system and the reflective (pre-frontal cortex) system [6,7,8]. Examples of these “dual-process” models include that of the incentive-sensitization theory [9], which suggests that through classical conditioning, a substance stimulus would increase in salience and result in increased motivation to acquire that substance (Robinson & Berridge, 1993 as cited in [9]). Therapies like cognitive behavioural therapy targets typically targets the reflective system. As aforementioned, the predominance of the impulsive (limbic) system and its associated biases might predispose individuals to a relapse. Biases common in addictive disorders include that of attention bias, which refers to the tendency for substance-related cues to selectively capture individuals’ attention in their naturalistic environment [9,10,11] and that of approach and avoidance biases, which refers to automatic tendencies to reach out for substance related stimuli (Wiers, R.W. 2013) [12]. To date, several studies have looked for attention biases in addictive disorders and the effectiveness of bias modification [12,13]. Cristea et al.’s (2016) [12] meta-analytic review involved studies that included participants with alcohol or tobacco use disorders, and reported that bias modification was effective, with an effect size (Hedges G) of 0.60. However, the changes in biases did not result in improvement in any other outcome measures, such as that of cravings. Although Cristea et al.’s (2016) [13] review provided evidence for the presence of biases in addictive disorders and the effectiveness of bias modification, the review was limited to only these two forms of substances of abuse. The included studies also had a high risk of bias. Christiansen et al. (2016) [14] on the other hand included in their review studies with participants who were using alcohol, tobacco, cocaine, or cannabis. Christiansen et al.’s (2016) [14] study included participants with a range of addictive disorders and demonstrated the presence of attention bias in these disorders. They reported that the evidence for the association between attention bias and relapse to be mixed and reported that the differences in the tasks and the limited sample size of the included studies could have potentially accounted for their findings. Whilst Christiansen et al.’s (2016) [14] review included participants with a range of addictive disorders, this review had limitations, in that the inclusion and exclusion crtiera were not clearly specified and the time frame in which the search was conducted was not specified. Furthermore, the databases searched included only PubMed, Scopus, and prior published reviews, but not PsycINFO and MEDLINE.

Substance disorders, such as that of opioid, cannabis use, and stimulant use, have been highly prevalent, based on the recent reports released [1]. The estimated annual prevalences for these disorders in 2015 were of 3.8%, 0.7%, and 0.77%, respectively [1]. In 2015, there have been an estimated 183 million, 35 million, and 37 million past-year users of cannabis, opioids, and amphetamines, respectively, and the numbers of these users far supersede that for other substances, such as that for ecstasy (22 million past-year users). Given this, further research involving these three highly prevalent disorders is of importance. Taking into consideration the limitations of the previous review and the number of new studies published that have evaluated bias modification for these highly prevalent disorders, there is thus a need for an updated systematic review to synthesise the information from these studies. The primary aim of this current systematic review is to synthesise the current evidence with regard to attention bias amongst opioid use, cannabis use, and stimulant use disorders. The secondary aim is to determine the efficacy of attention bias modification interventions including attentional bias reduction and other addictions related outcomes. 

## 2. Methods

In our current review, we adhered to the plan as set out in our published protocol [15]. To achieve the objectives of the current review, a search was conducted from November 2017 to January 2018. The following databases were searched from inception―PubMed, MEDLINE, Embase, PsycINFO, Science Direct, Cochrane Central, and Scopus. The following search terminologies were used, such as (“attention bias” or “approach bias” or “avoidance bias” or “cognitive bias”) and (“addiction” or “substance” or “drug” or “abuse” or “dependence” or “opiates” or “heroin” or “cannabis” or “marijuana” or “stimulants” or “amphetamines” or “cocaine”). 

### 2.1. Inclusion and Exclusion Criteria

Articles written in English language were included. Articles were also included if they fulfilled the following inclusion criteria: (a) attention bias which was assessed using a validated measure; (b) participants in the studies must have a primary diagnosis of opiate use, cannabis use, or stimulant use disorder; and (c) the study design is a randomised trial. Articles were excluded if (a) they did not include a validated measure for the assessment of attention bias; (b) participants in the study were diagnosed with other psychiatric disorders as the primary diagnosis (for example, Depression or Anxiety Disorders); (c) studies involved a pharmacological intervention in which medications were utilised to examine their effects on attention bias; or (d) the randomised trial involved a cross-over design. For (d), these randomised trials were excluded due to their high risk of bias. 

Considering the limitations of prior reviews, only randomised trials were included for the current review. 

### 2.2. Selection of Articles

Selection of relevant articles were conducted independently by two authors (Melvyn Zhang and Jiangbo Ying). Articles were first screened based on their titles and abstracts. Articles that were shortlisted were then evaluated against the inclusion and exclusion criteria. In the event of any disagreements with regard to the screening of the articles based on their titles and abstracts, or if there was no consensus whether to include or exclude the article, this would be discussed and resolved with the author (Guo Song).

The selection process was in accordance with the Preferred Reporting Items for Systematic Reviews and Meta-Analysis guidelines [16]. 

### 2.3. Statistical Analysis

#### 2.3.1. Extraction

The following data and information was extracted from each article and recorded on a standardised electronic data collation form: (a) Publication details (author(s) and study year); (b) Study design and methodology (Study design, sample size, types of sample, country, demographics of sample, diagnosis of participants, methodology in which diagnosis is made); (c) Attention bias assessment and modification methodology; (d) Outcomes of interest (e.g. craving scores, addiction outcomes, effect size for attention bias modification procedure). 

#### 2.3.2. Data Analysis and Synthesis

Due to the diversity of the outcome measures reported, meta-analytical synthesis was not feasible and a qualitative synthesis was undertaken instead. 

#### 2.3.3. Quality Assessment

The Cochrane Risk of Bias tool was used for the risk of bias assessment. Risk of bias assessment was conducted for the following domains: selection bias, performance bias, detection bias, attrition bias, reporting bias, and other bias.

## 3. Results

**Figure 1:**
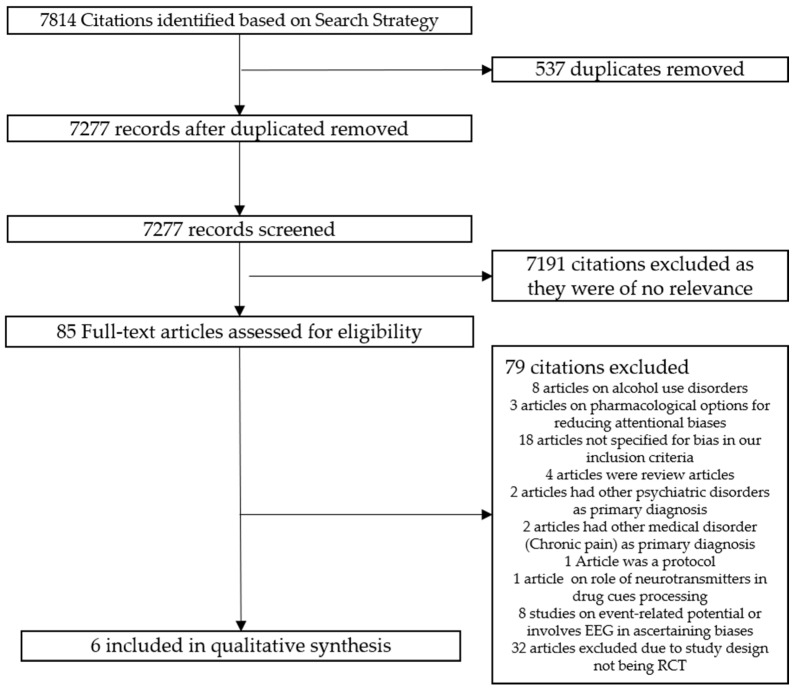
Flowchart for the Selection of Articles.

Based on our pre-defined search strategy, a total of 7814 citations were identified from the databases; 537 duplicated articles were excluded, 7277 records were screened, and 7192 citations were excluded as they were of no relevance to the topic of interest. A total of 85 full-text articles were downloaded for further evaluation against the inclusion and exclusion criteria. Seventy-nine citations were excluded as they did not fulfil the inclusion criteria, leaving six articles for the eventual qualitative synthesis. Figure 1 provides an overview of the study selection process. Table 1 provides an overview of the characteristics of the included studies. Half of the identified articles involved cohorts of individuals with opiate use disorder and the other half of the identified articles included cohorts with cocaine use disorder.

### 3.1. Overview of Studies

Of the six identified randomized trials, only one study [21] investigated specifically attention bias modification on attention bias. Franken, I.H. et al.’s (2002) [17] study involved having participants undertake a supra- and subliminal Stroop task for the assessment of attention bias. Similarly, in Lubman, D.I. et al.’s (2000) [18] trial, the pictorial probe task that was used for the assessment of attentional bias. Marissen, M. et al.’s (2006) [19] trial investigated the effects of cue exposure therapy, instead of attention bias modification on attention biases. In the remaining two cocaine trials, one trial (Montgomery et al.’s (2010)) [20] evaluated the effects of alcohol administration on attentional biases. DeVito, E.E. et al. (2017) [22] in their study evaluated the effects of computer-based CBT (Cognitive Behavioral Therapy), as it is believed that CBT could strengthen cognitive control processes. 

### 3.2. Characteristics of Three Studies for Opioid Use Disorders

The three randomised trials that included individuals with opiate use disorders recruited these individuals from either an inpatient treatment unit, local drug and treatment services, or individuals who admitted themselves into a drug and rehabilitation therapeutic unit for treatment. The sampled individuals were all from treatment seeking individuals. With respect to demographics, the mean age of the included participants was in their thirties, and all three involved a predominantly male cohort. The diagnosis of the individuals was all ascertained using psychiatric assessments, based on either the DSM-IV criteria or the ICD-10 diagnostic criteria. Two out of the three trials utilised Stroop-based task in the assessment of attention bias (Franken, I.H. et al., 2000; Marissen, M. et al., 2006) [17,19]. 

### 3.3. Primary and Secondary Outcomes Reported in Three Studies for Opioid Use Disorders

All three trials reported the presence of attention biases in individuals with opioid use disorders. Franken, I.H. et al. (2000) [17] reported that individuals with opioid use disorder had longer reaction time for cues as compared to controls, and Lubman, D.I. et al. (2000) [18] also reported that individuals tend to demonstrate faster reaction times for probes that replaced drug-related stimuli. There were varied secondary outcomes reported in each of the included studies. Franken, I.H. et al. (2000) [17] reported that bias modification led to a decrease in the cravings measure. Marissen, M. et al. (2016) [19] reported that baseline attention biases were predictive of subsequent relapses at three months follow-up. Marissen, M. et al. (2016) [19] also reported that cue exposure therapy did not lead to a significant reduction of attentional biases as compared to placebo psychotherapy. 

### 3.4. Characteristics of Three Studies for Stimulant Use (Cocaine) Disorders

Only two out of the three trials provided information about the source of their participants. There was diversity in that Montgomery et al.’s (2010) [20] trial, recruited a student sample, whereas DeVito, E.E.’s (2017) [22] trial recruited participants from the community-based clinics. The mean age of the sample also varied, ranging from a mean of 19.29 years to that of 42.2 years. Males did not predominate in the trials. It is important to note that the diagnosis of cocaine dependence was established using a questionnaire for Montgomery et al.’s (2010) [20] trial, whereas the diagnoses for the remaining trials were ascertained by mean of the structured clinical interview and the DSM-IV criteria. Both visual probe task and Stroop task were used in the ascertainment of attention biases. 

### 3.5. Main Outcomes Reported in Studies for Stimulant Use (Cocaine) Disorders

All three trials reported the presence of attention biases in individuals with cocaine use disorders. Similarly, the three trials also involved interventions to modify attentional biases. Montgomery et al. (2010) [20] demonstrated that attention biases towards cocaine stimuli were heightened when alcohol was administered, whilst DeVito, E.E. (2017) [22] reported that attentional biases reduced with the intervention. Notably, Mayer, A.R. et al. (2016) [21] reported in their trial that attention bias modification was not effective in reducing biases. In the three included randomised trials, only Mayer, A.R. et al. (2016) [21] evaluated the efficacy of attention bias modification. DeVito, E.E. (2017) [22] evaluated the potential of computer-based CBT in enhancing executive cognitive control processes and reducing attentional biases. 

Using the search strategy, we were unable to identify any randomised trials for cannabis use disorders.

### 3.6. Quality and Risk of Bias Assessment

**Figure 2:**
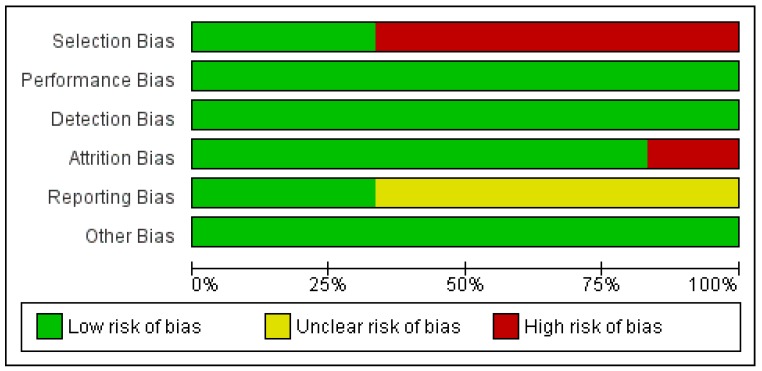
Risk of Bias Assessment for included studies.

**Figure 3:**
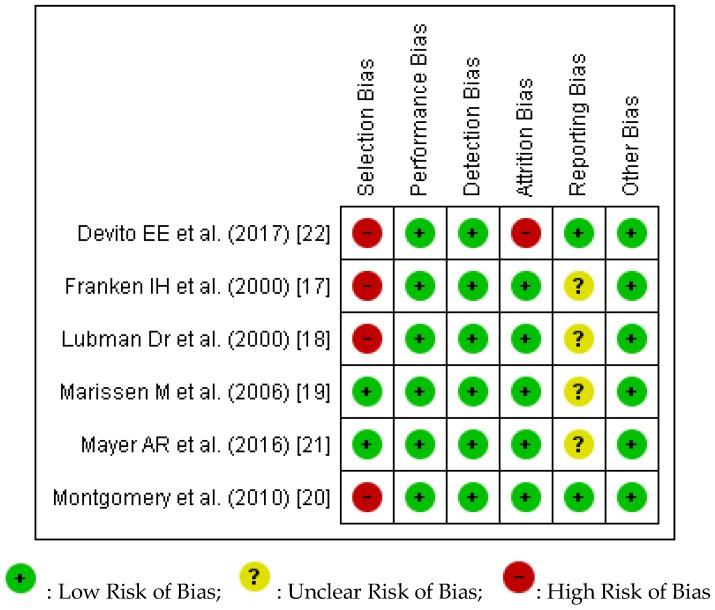
Risk of Bias assessment for each of the included studies.

We conducted a risk of bias assessment of the included studies using the Cochrane Risk of Bias tool. Review Manager 5 (RevMan5) (Cochrane Collaboration, 2014) [23] was used to prepare both Figure 2 and Figure 3. Figure 2 provides an overview of the overall risk of each type of biases for all the studies. The percentages of low, unclear, and high risk of biases were automatically computed by RevMan5. Figure 3 provides an overview of our assessment of the risk of biases in each study. 

Across all six studies, there was demonstration of high risk in both selection bias and attrition bias. Franken, I.H. et al.’s (2000) [17] study was graded as being high risk for selection bias due to the nature of its recruitment (inpatient treatment centre for participants and clinics for controls) and the discrepancies in the level of education, with controls having higher levels of education. Similarly, Lubman, D.I. et al.’s (2000) [18] trial included heroin participants who were noted to have significantly lower intelligence quotient and higher levels of anxiety and depression as compared to the controls. Selection bias was also present in Montgomery et al. (2010)’s [20] study, in that cocaine users had significantly higher scores on the AUDIT (Alcohol Use Disorder Identification Test) as compared to the non-users. DeVito, E.E. (2001) [22] study also rated high on the risk of selection bias as only participants with Stroop data at pre and post assessment were included. DeVito, E.E. ’s (2001) [22] trial also had a high risk of attrition bias, as out of the original 101 participants recruited, data computation and analysis were only performed on an eventual sample of 61.

## 4. Discussion

Our current review has managed to identify more randomized trials than Cristea et al. (2016)’s [13] prior meta-analytical review for opioid and cocaine use disorder. Six randomised trials were identified to be included in the current systeatic review, with three trials involving participants with opioid use disorder and three trials involving participants with cocaine use disorders. The evidence synthesized from these trials have provided strong evidence that attention biases are present in opioid and stimulant use disorders. There was similarity in the demographics of the participants of the three trials with opioid use disorder, in that the sample were treatment-seeking individuals, predominantly male, and the diagnosis was ascertained based on psychiatric assessments. Across all six trials, attention bias was reported to be present, despite the different methods of assessment with either the use of Stroop test or Visual probe task. Across the six trials, there remained no consensus that attention bias modification was effective. Evidence synthesis for other secondary outcome measures could not be performed given the heterogeneity in the measures reported and the limited number of trials. The risk of bias assessment for the included trials revealed a high risk of selection and attrition bias. 

Our current review, by synthesizing the evidence from randomized trials, has provided evidence that attention biases are present in both opiate-use disorders and stimulant (cocaine) use disorders. The current findings complement and expand on the findings that Cristea et al. (2016) [13] previously reported. As aforementioned, their meta-analytical review included 24 studies that involved alcohol use and tobacco use participants. Even though the prior review made use of a search strategy that included keywords such as “cannabis, marijuana, cocaine, heroin, opiates, amphetamines”, randomized trials were not identified and hence evidence synthesis was limited to alcohol and tobacco addiction only. We acknowledge that in Christiansen et al.’s (2015) [14] review, they have identified more studies than in our reiew. They have identified three studies involving heroin-dependent participants and five studies involving cocaine-dependent participants. However, as aforementioned, the authors did not specify their inclusion and exclusion criteria and failed to utilize a systematic review approach and this limits the quality of the evidence synthesized. Nevertheless, their findings help to demonstrate that attentional bias are present in these disorders and are evident across a variety of study designs. In summary, our results fulfills the gaps in knowledge of Cristea’s prior meta-analysis and add on to the evidence synthesized in Christiansen et al.’s (2015)’s review [14]. Attention biases are present in opioid and cocaine use use disorders, and this finding is of clinical importance, as if attentional biases are present, specialized interventions could be devised to help retrain these automatic biases.

The results arising from the opioid trials demonstrate the presence of attention biases in individuals who were maintained on opiate substitution therapy (Lubman, D.I. et al., 2000) [18]. The presence of attention biases amongst individuals maintained on opiate substitution therapy implies that, whilst pharmacological interventions might help in the stabilization of lifestyle and minimization of harms associated with illicit usage, psychological interventions such as attention bias modification needs to be considered as augmentative treatment, to help address the automatic processes that might result in a relapse. Franken, I.H. et al. [17] proposed in his review that drug-related stimuli is known as “cognitive intermediates” prior to a lapse or relapse, given that such salient stimuli activates unconscious processes leading to one having increased attention, but also leaving the individual with fewer resources available to apply learned coping strategies. Hence, there is clearly a need for attention bias modification to be considered for individuals with addictive disorders. There has also been increased recognition of this need, as evidenced by Heitman’s et al. (2017) [24] proposed protocol, in which they attempted to investigate both the effectiveness and cost-effectiveness of an online, Internet-based attention bias modification delivered in complement to usual intervention for individuals with alcohol or cannabis use disorders. Whilst there was a prior study (Hullu et al., 2017) [25] that evaluated combined cognitive bias modification and cognitive behavioural therapy, there remains, to our knowledge, no similar published studies or protocol for substance use disorders. A consideration of the integration of both modalities of therapy is crucial, given that cognitive behavioural therapies typically target the top down or reflective conscious decision-making processes, whilst bias modification targets the bottom-up or unconscious processes that could be responsible for lapse and relapse.

Amongst the studies identified for the current synthesis, only one study evaluated attention bias modification. The remaining studies evaluated other forms of intervention, including that of cue-exposure therapy and computerized cognitive behavioral intervention. Given this, this limits the evidence synthesis for the overall effectiveness of bias modification. Notably, most of the included studies were studies that merely evaluated for the presence of attention biases using mainly the Stroop task (in four studies). The remaining studies evaluated for attention biases using the visual probe task. Since different interventions were used, it is hard to compare whether the different tasks used (Stroop versus the visual probe) did have an effect on the overall results.

There are many strengths of this current review. We have comprehensively searched through the literature for randomized trials involving highly prevalent addictive disorders. Our current review identified more trials than Cristea et al.’s (2016) [13] review for opioid and stimulant use disorders. This is because we have searched through additional databases, namely that of MEDLINE, ScienceDirect, and Scopus, and the search was conducted up until 2017. Through our extensive search strategy, we managed to include trials that have been conducted among participants with highly prevalent substance use disorders, specifically that of cocaine use disorder. The findings arising from the current review help to provide evidence for the presence of attentional biases in other addictive disorders, and this is important clinically. It is important for clinicians to recognize that automatic processes such as attention biases could predispose individuals with opioid and cocaine use disorders towards a lapse or relapse. Clinicians need to recognize that for the chronically addicted individuals, psychological interventions such as cognitive behavioural therapy for relapse might not be adequate and there is a potential role for augmenting that treatment with attention bias modification. We managed to perform a risk of bias assessment to assess the quality of the trials that were included. We have adhered to our planned protocol and used a predefined search strategy and have adhered to the specified inclusion and exclusion criteria. 

Despite the strengths, there are also several limitations. In our current review, we only included six trials. Our main limitations pertain to the fact that there might be other studies that have been published and were evaluated for the presence of attentional bias and the effectiveness of attentional bias modification but were excluded due to their study designs. We have not in our current review included the articles that Christiansen et al. (2015) [14] included in their prior review, as the prior review included studies with different study designs. We have originally considered in our review only randomised trials for inclusion in order to enhance the quality of the evidence derived by the synthesis [26]. There are intrinsic advantages with regard to considering only randomised trials. Trials involving randomisation remove the inherent chance of confounding, and the double-blinded process also helps in the minimisation of biases (Shrier, I. et al., 2007) [26]. However, Shrier, I. et al. (2007) [26] previously articulated the need to consider the inclusion of other trial designs as the additional information derived could help in clinical reasoning and provide a solid foundation for causal inferences. Similarly, Peinemann, F. et al. (2013) [27] also opinionated that the consideration of the types of studies to be included is dependent on the clinical question that needs to be answered. Thus, for our current review, the main clinical question relates to whether attentional biases are present in highly prevalent substance use disorders, and whether these biases could be modified, and to look at its effect on other secondary outcome measures. As such, restricting the inclusion criteria to include only randomised trials would result in a significant number of observational studies being left out, as demonstrated by Christiansen et al. (2015)’s [14] review. Moreover, whilst attention biases are widely studied for alcohol and tobacco use disorders, attention bias and bias modification for stimulant use, cannabis use, and opiate use disorders are less well characterised. Also, given that many of these substances are considered illicit globally, it is potentially difficult for researchers to conduct randomised trials, given the ethical and legality concerns associated with a randomised trial. Given the high risk of bias in some of these randomised trials, especially for attrition and selection bias, randomisation has potentially not minimized the risk of confounders. Hence, the quality of the evidence synthesised from these limited randomised trials might not be superior in comparison to a review that considered the inclusion of a variety of study designs. Hence, in retrospect, to better answer the research and clinical question, a consideration of different study designs might yield more evidence for attentional bias modification in this emerging field, instead of limiting just to randomised trials. In addition, our review is limited as it did not include any studies involving participants with cannabis use disorder, which is a highly prevalent disorder globally. Lastly, we were limited to a qualitative synthesis of the data extracted from each of the studies, and a meta-analytical synthesis was not appropriate due to the heterogeneity in the outcomes reported. 

## 5. Conclusions

The findings from our current review demonstrate the presence of attentional biases in two highly prevalent addictive disorders, that of opiate use and cocaine use disorders. Due to the heterogeneity in the secondary outcomes reported, the current review is unable to provide evidence pertaining to the effectiveness of bias modification, and the effect that bias modification has on other secondary outcomes. Nonetheless, this review is important as it demonstrates the potential need for interventions targeting attention biases in opiate and cocaine use disorders. Future research should consider the inclusion of other study types to better synthesise the evidence for attention bias and bias medication in these highly prevalent addictive disorders. 

## Figures and Tables

**Table 1:** Characteristics of Included Studies (*n* = 6).

Study	Study Design	Sample Size	Types of Sample	Demographics of Sample	Country	Diagnosis of Sample	Method of Diagnosis	Attention Bias Method	Outcomes
Franken, I.H. et al. (2000) [17]	Randomised trial	21 heroin-dependent participants 30 control participants	Participants with heroin dependence were recruited from an inpatient treatment centre Participants in the control group were recruited among clinical and administrative staff in the clinic	71.4% males (heroin-dependent) 83.3% (control group) Mean age 31.5 years (heroin-dependent) Mean age 34.8 (control group) Mean self-reported duration of heroin dependence was 93.9 months	Netherlands	Heroin Dependence	Based on the DSM-IV criteria for heroin dependence	Drug Stroop task	Higher overall reaction time for heroin participants as compared to control participants Mean pre-experimental craving was 13.8, mean post masked Stroop was 7.19 and mean post unmasked Stroop was 15.2
Lubman, D.I. et al. (2000) [18]	Randomised trial	16 methadone-maintained opiate addicts 16 age-matched control	Heroin addicts were recruited from local drug services Staff from these services were recruited as controls	Opiate group: mean age 31.4, Male to Female 11:5 Control group: mean age 31.7, male to female 8:8	United Kingdom	Heroin Dependence	ICD-10 and DSM IV	Pictorial Probe Detection Task	Faster reaction times to probes that replaced drug stimuli, indicative of the presence of an attentional bias
Marissen, M. et al. (2006) [19]	Randomised trial	110 Participants assigned to either cue exposure therapy or placebo psychotherapy	Abstinent heroin addicts who were admitted voluntarily to an in-patient drug-free therapeutic centre in the Hague	89% males, mean age 34 years old Average age of onset of heroin usage was 21.4 years, most have used heroin for 9.3 years	Netherlands	Heroin Dependence	DSM-IV criteria for heroin dependence	Emotional Stroop Task	Pre-treatment attentional bias predicted relapse at three months follow-up Reduction of attentional biases in both experimental conditions
Montgomery et al. (2010) [20]	Randomised trial	32 regular cocaine users and 40 non-users	Student Population at Liverpool John Moores University and the general population in the surrounding areas	Mean age for cocaine users assigned to placebo 19.29, assigned to alcohol 20.23 Mean age for non-users assigned to placebo 19.59, assigned to alcohol 20.0 13 male in cocaine use group 19 male in non-cocaine use group	United Kingdom	Cocaine dependence	Questionnaire	Visual Probe and Modified Stroop task	Cocaine participants who received alcohol had increased attentional bias for cocaine pictures The cocaine Stroop revealed no differences between cocaine users and non-users, and no effects of alcohol in either group
Mayer, A.R. et al. (2016) [21]	Randomised trial	37 participants assigned to either attentional bias modification therapy (ABMT) or control therapy	Not mentioned	ABMT group: 14 male, and 5 female, mean age 37.4 Control group: 10 male and 8 female, mean age 38.9	United States	Cocaine dependence	Structured clinical interview for DSM-IV	Visual Probe task	Presence of attentional bias Attentional bias not subjected to modification by ABMT
DeVito, E.E. (2017) [22]	Randomised trial	38 in treatment as usual plus computer-based CBT (CBT4CBT) 41 in treatment as usual	Recruited from community-based outpatient clinic	46% female, mean age 42.2	United States	Cocaine use disorder	DSM-IV	Computerized drug Stroop test	Stroop testing revealed that participants who have had a longer duration of cocaine abstinence during treatment (3+ weeks) have greater reductions in Drug Stroop effect. Engagement with CBT4CBT intervention also led to a reduction in Drug Stroop effect
